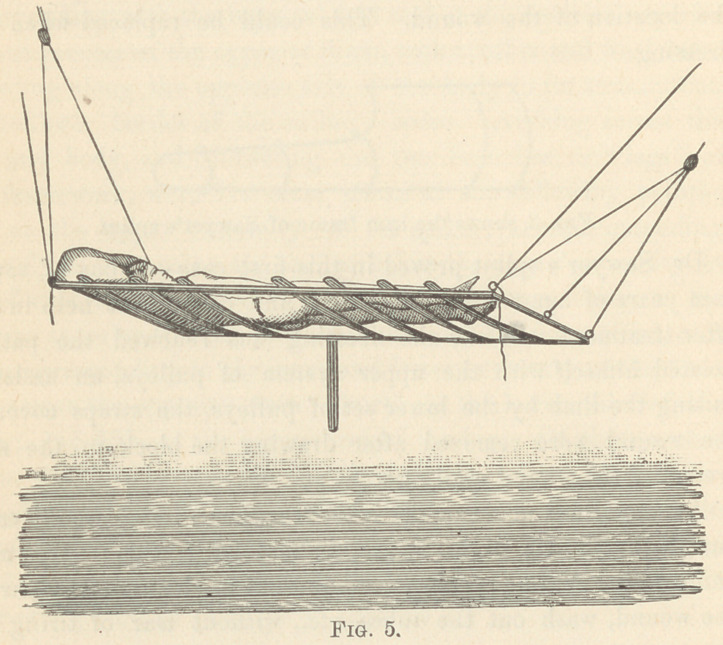# Select Topics of Modern Surgery, Illustrated by Cases from Hospital Service and Private Practice

**Published:** 1880-07

**Authors:** E. W. Lee, Chr. Fenger

**Affiliations:** Chicago; Chicago


### Original Communications.

ARTICLE II.

SELECT TOPICS OF MODERN SURGERY, Illustrated by Cases from Hospital Service and Private Practice. By Drs. E. W. LEE and CH. FENGER, of Chicago.

### TUBERCULOSIS OF JOINTS, WITH THREE CASES OF EXCISION.

*Tuberculosis of the knee-joint. Excision by Dr. Ch. Fenger.*

The discussion of the subject of exsections of the knee-joint which follows, is abridged from a clinical lecture delivered in Cook county hospital, where the operation was made.

Exsection of the knee-joint is generally considered a serious, not to say dangerous, operation. We shall now first consider this point; then its indications in general and in this case; and finally the methods of operating which this case is intended to illustrate.

For the danger to the life of the patient we must seek information in the statistics.

A series gathered by König (Beiträge zur Resectionen des Kniegelenkes. *Langenbeck's Archiv.* ix B. p. 177, and Holmer Hospitalstidende Optegnelser of praktisk Lägekonst, 23 October, 1872) is as follows:

| *Surgeon.* | *Good Result.* | *Failure.* | *Mortality.* |
|---|---|---|---|
| Hodges | 44 per cent | 56 per cent | 33 per cent. |
| Holmes | 62 " | 38 " | 28 " |
| Heyfelder (children and adults) | 60 " | 39 " | 30 " |
| Heyfelder (adults) | 44 " | 56 " | 39 " |
| Price | 56 " | 43 " | 27 " |
| König (children) | 62 " | 37 " | 19 " |

These statistics were gathered about 1870, and show a mortality of about 30 per cent. This is the death rate of the statistics of Péničres (Des resections de genoux Paris, 1869), who found in 431 cases of excision of knee-joint for white swelling, 131 deaths, that is 30 per cent.

From English surgeons, however, we have smaller series of operations, with far better results.

| | | | | |
|---|---|---|---|---|
| Fergusson reports in an old series | 31 | cases | with 11 | deaths. |
| In a later series | 20 | " | " 5 | " |
| Humphrey reports | 39 | " | " 6 | " |
| Jones reports | 19 | " | " 2 | " |

In considering the value of the statistics there are two interesting facts.

1. The age of the patient has great influence on the death rate, as shown by Péničres :

| *Age.* | *Death Rate.* |
|---|---|
| From 0 to 5 years | 38.8 per cent. |
| " 5 to 10 " | 15 " |
| " 10 to 15 " | 18.9 " |
| " 15 to 20 " | 32.7 " |
| " 20 to 25 " | 35.7 " |
| " 25 to 30 " | 37 " |
| " 30 to 40 " | 45 " |

In one series of Péničres were 30 excisions in children from nine to eleven years without a death. The death rate is lowest therefore in children from five to about 20, and the danger as the age advances gradually increases.

2. The period in which the operation was performed is next in importance, insomuch that we find the death rate from the early days of this operation by surgeons to a recent date to be steadily decreasing. Péničres gives the following statistics on this point:

| | | | | |
|---|---|---|---|---|
| From 1762 to 1830 there were | 11 excisions, | 6 deaths. | Per cent., | 54.5. |
| " 1838 to 1850 " | 21 " | 11 " | " | 52.3. |
| " 1850 to 1860 " | 246 " | 73 " | " | 27. |
| " 1860 to 1869 " | 155 " | 42 " | " | 27. |

Better methods of operating, better after-treatment and better knowledge of the indications for the operation may account for this decrease in death-rate. But even in 1873 the average death rate was not below 27 per cent. Exceptions were Fergusson's second series, 25 per cent.; Humphrey's series, 15 per cent.; and Jones' series, 10.6 per cent.

In Germany up to this time (in 1873) the death rate is considered by Volkmann to be about 50 per cent. (Sammlung Klinisher Vorträge, R. Volkmann, Die Resectionen der Gelenke, 1873). Very naturally, therefore, Volkmann preferred the expectant treatment in white swellings of the knee-joints in the absence of fever and wasting discharges that threaten the life of the patient.

The next and latest important step toward a more favorable result for this operation is Lister's method of operating and dressing.

In 1872 Holmer of Copenhagen made four excisions for white swelling, with three good results, one secondary amputation and no deaths.

In 1875, '76, '77 Volkmann made thirty-two excisions for the disease with no deaths from the operation. One patient died several weeks later of tubercular meningitis.

It is now impossible to know the exact danger to life from this operation. We cannot expect to reduce the death rate to zero by antiseptic methods, for that would be perfection. We can only hope to come nearer and nearer the ideals without ever reaching them. But the latest statistics from other fields of operative surgery give us the right to expect that excision of the knee-joint will henceforward be an operation devoid of very great danger whenever all its details and after-treatment are performed with strict antiseptic precautions. But the surgeon must have patience to attend to all the little details of the antiseptic method, on each of which the life of a patient may depend.

The object of excision of the knee-joint is obvious. It is to save a limb that otherwise must be lost sooner or later by amputation, this latter operation being finally required in cases where recovery is hopeless by abatement of the inflammation or by such firm anchylosis in a false position as will not give the patient a useful limb.

By this operation we create a firm anchylosis which the patient can use continuously without pain or fatigue.

The question now is, can we accomplish this purpose of the operation without months of confinement and suffering in bed for the patient, with such abundant suppuration as would endanger his life from exhaustion, amyloid degeneration of the kidneys, liver and spleen, even if he should escape pyæmia?

From Hodges' statistics, Gant puts the average time of after-treatment at eight months. This is a long time if the patient must remain in bed and sustain a constant suppuration.

Fortunately this is not the case. As soon as a solid osseous union has taken place between the cut ends of the femur and tibia we may allow the patient to be about on crutches. Fergusson has had patients up in this way in three to six weeks after the operation; but such cases are regarded as fortunate exceptions. Holmer had his patients up and on crutches after three or four months.

Under the Lister dressing suppuration is sometimes reduced to a mere trifle, generally to a moderate amount; so there is no danger from exhaustion.

If then, the patient can expect to be up after an average of three months and be sure of a useful limb after other three or six months, he is far better off than he could be without the operation.

The history of this case is as follows:

James C., æt. 15 years, clerk (in chair factory), entered Cook County Hospital July 15, 1879. None of his relations have suffered from consumption or cancer. Had measles and typhoid fever in childhood; always healthy since. Two years ago he received a kick from a playfellow upon his left knee. Pain disappeared in a few days and there was no swelling. Three or four months later the knee-joint began slowly to swell and be occasionally painful; and motion became gradually impaired. The joint felt to him stiff. The knee remained in this condition about six months, during which time he could walk, run and jump without much pain. Then one day, when jumping, he suddenly felt severe pain in the joint and he was obliged to sit down two hours. The pain gradually subsided and soon he was able to run about again for a short time. But gradually the swelling increased, pain came on and he got easily tired.

About a year ago abscesses formed and opened on the outer and inner sides of the joint near the hamstring tendons. Fistulæ

were left which discharged six months; they closed to break open later again and again close up. No spiculæ of bone were discharged to his knowledge.

Contracture now came on and the leg was at last flexed at nearly a right angle and motion was limited to twenty degrees. In spite of this condition he could, most of the time, hobble about, bearing some weight on the leg; but the leg easily tired, and on stepping upon an uneven surface or jarring the knee, pain was produced. He is constanly afraid to have any one come near the limb for fear of hurting it.

On July 3d (12 days ago), while at work, a sudden pain came on and the knee got worse. Now any considerable motion causes pain. In perfect quiet pain is absent. As to previous treatment he says at one time a doctor tried to aspirate the joint, getting no fluid. Another time a blister was applied over the joint with the effect of ameliorating the condition.

He is now slender, pale and lean. The heart, lungs and abdominal organs are healthy. The urine has no sugar or albumen. The left knee is enlarged forming a prominent round tumor. The joint is flexed to about 110 degrees and motion is allowed of only 10 degrees; attempts to increase the range of this causes pain. The relative position of the crus to the thigh at the joint is that of genu varum, knock-knee. This means a partial dislocation due to weakening or partial destruction of the internal lateral ligaments. On the sides of the joint are depressed reddish spots, the seats of the closed sinuses. The patella is immovably fixed to the fossa of the condyles of the femur. The swollen soft tissues form a uniform softish somewhat elastic mass, like India rubber of medium hardness. There is no fluctuation or other evidence of fluid in the capsule or outside the joint. Pressure upon the mass does not cause pain except at two points on the sides corresponding to the spaces between the joint surfaces covered by the lateral ligaments.

We have here a chronic fungous arthritis, or white swelling.

The constituents of the joint are already partially destroyed as proves the adherent patella and weakened internal ligaments and the partial anchylosis, i.e. the impaired motion.

We have now to ask what would be the result of this case without operation.

The destructive inflammation here has been slowly progressing; motion has grown less and less, partly from tenderness and pain, partly from false position. We have for the three weeks he has been in the hospital tried gradual extension (by weight and pulleys) to correct the flexion, and hot fomentations for the pain. The treatment has not had the slightest effect. We then have left the choice between forcible extension of the joint under anæsthesia, and immobilization with plaster of Paris, or starch, and excision.

The liability of the inflammation at the bottom of the old sinuses to be lighted up anew speaks against immobilizing bandages. They would have to be removed at intervals. This would make recovery by anchylosis in good position unlikely to occur. This treatment would probably be interrupted by abscesses that might extend up and down between the muscles and make amputation necessary, and excision, if it was to be made, much more uncertain of a good result.

The age of the patient is in favor of excision, and a solid union of the bones with diseased soft parts removed is decideaiy preferable to a more or less complete anchylosis with diseased tissues remaining between and around the epiphysis of the bones, because in this case there would always be a liability to a relapse of the inflammation.

Excision being then decided on, the next question is the plan of operating.

1. Esmarch's bandage for operating without bloodshed should be used when there is no danger of pressing pus or infectious thrombi of the veins, up into the healthy soft parts above the seat of operation. As there are no abscesses and no œdema we shall use it here. Besides avoiding blood loss, this method enables us to distinguish the character of tissues with much more exactitude than we could without it. It is not only consistency but color that indicates what tissues are to be removed, and what not. The well-known light yellowish gray color of cheesy matter means fatty degeneration of the tissue—that it is dead or dying and must be removed.

2. The incision that gives the easiest access to all the different parts of the joint is the semilunar cut commencing at the tuberosity of one of the condyles of the femur, a right handed operator will commence it at the internal condyle of the right knee and the external of the left, coming down toward the tuberosity of the tibia and returning to the other condyle. It divides the inferior patellar ligament and subjacent adipose tissue. These parts lifted up give an easy access to the joint.

3. After division of the lateral and cruciatic ligaments a forcible flexion of the joint will show us the condition of the joint and enable us to bring the epiphyses to turn out so that the diseased part may be sawed off. For this purpose we use Butchers' saw and cut the femur from the joint backward.

As to the line of the cut, if this is parallel to the line uniting the lowest points of the two condyles, we will remove too much of the external condyle and get after coaptation a position of knock-knee. If we cut in a line perpendicular to the longitudinal axis of the femur we remove too much of the internal condyle and the opposite deformity is the result i.e., bow-legs. Linhard advises to cut just between the two lines described. Of the epiphysis of the tibia we remove a disk, cutting from the posterior surface anteriorly and parallel to the articular surface.

As to the thickness of the disks to be removed, we should remove all the bone diseased but not a particle more. We should endeavor furthermore to have two even surfaces of equal size of bone that perfect union by first intention may if possible take place.

In children the epipyseal line has a certain importance for the future growth in length of the bones of the limb. If possible we should spare the whole or a part of the epiphyseal cartilage. Anatomical investigations by Giraldez place the limits beyond which we are not to go as follows: In the condyles of the fermur, 2 cm., in the intercondyloid fossa, 1.5 cm., in the tibia 1.5 cm. Still more exact details are given by König (*Langenbecks Archiv.* i. x.). The epiphyseal line is situated in a child of 11 as follows (from the articular surfaces,) internal condyle of femur 2.4 cm.; external condyle 2.1 cm.; anterior portion of intercondyloid fossa 1.6 cm.; posterior part 1.4 cm.; anterior part tibia, (near tuberosity) 3.8 cm.; posterior part 1.5 cm.; medium inner part 1.5 cm.; external lateral half 1.4 cm.; Each additional year adds one millimeter to the depth of the epiphyseal cartilage. In the present case we shall cut off 2 cm. of the femur and ½ to 1 cm. of the tibia, and then if the surfaces are healthy unite them; if local deseased foci are present scoop them out with the gouge rather than remove a larger portion of the epiphyseal cartilage. Should the whole epiphysis, clear to the medullary cavity be diseased we may have to amputate.

The patella, if diseased, is to be removed. If not, shall it be left? From Pénières statistics we learn that saving the patella raises the death rate 30 per cent. and more than doubles the chances for the necessity of a secondary amputation. Hodges calculates that the removal of the patella shortens the after-treatment 30 days. Holmer makes the sensible remark that the patella, if left in its place, makes the internal cavity of the wound irregular, thus making pockets for fluids that should be discharged and besides presents posssible starting points for new caries. Therefore we shall remove the patella in this case.

4. Excision of the thickened capsule is the next step. The whitish, firm or grayish elastic tissue into which the soft parts of the diseased joint are transformed, must be removed. Authors differ as to the advisability of this step. The majority advise the removal of what conveniently can be, and say that particles of indurated tissue left will do no harm but will be absorbed or will participate in the formation of the cicatrix. The minority advise the removal of every particle of the fungous capsule, even the posterior wall to the coats of the popliteal vessels. One author, Prof. Albert of Insbruck* advises the extreme step that we secure the popliteal artery and vein by a loop before dissecting away the adjacent capsular tissue, that no accident to these vessels may necessitate amputation. We shall, in this case, remove as much as possible of the thickened capsular tissue without resorting to the ligation referred to.

5. We next shall drill a hole through the bones on each side and pass in a soft silver wire of 0.5 to 1 millimeters diameter.

6. After loosening the elastic constriction of the arteries above the knee we shall stop hæmorrhage by ligatures with catgut of possible bleeding vessels, elevate the field of operation and wash it out with 2½ to 5 per cent. solution of carbolic acid.

---

* *Wiener Med. Presse*, No. 24, 1879. Beiträge zur Operativen Chirurgie.

7. A drainage tube will be placed between the posterior wall of the capsule and the posterior aspect of the cut osseous surfaces, i.e. the tube is carried along the whole of the posterior line of the united cut surfaces of the epiphyses.

8. We then unite the silver wire sutures so as to procure a uniform and perfect contact of the cut osseous surfaces.

9. The limb is then to be placed in a padded white metal splint, with movable doors at each side of the joint to admit of free application of the antiseptic dressings.

It is a Petits splint modified a little as shown in

FIG. 1.

The strings from the upper margin of the splint allow of the suspension of the whole limb. The pads consist of carbolized cotton covered by protective or carbolized oil silk.

This mode of bandaging procures immobilization and gives easy access to the wound, without disturbing it, for dressing.

The fenestrated plaster bandages are good for fixing the limb, but do not permit of renewing antiseptic dressing without soiling the bandage.

A plaster dressing cannot be kept clean enough to avoid danger of septic infection of the wound.

Having placed the limb in the padded splint and brought the osseous sufaces in perfect contact we insert another drainage tube along the anterior margin of the osseous union and unite the wound.

First we unite the divided inferior patellar ligament. Next we unite the wound with cat-gut or carbolized silk, leaving only openings for the silver wire and drainage tubes. The ends of the latter we may fasten to the skin by a stitch of silk suture and then cut them off short and obliquely, so as so facilitate the introduction of the point of a syringe for aspirating fluids and washing out with antiseptic solution if necessary.

The operation was made with the aid of Drs. Isham, Lee and

Jacobson of the Hospital staff, and Drs. Sawyer, Clausen and Murphy of the house staff.

The mode of operation was as already indicated. There was no purulent fluid in the joint. The capsule was firm, whitish and 3 to 5 millimeters thick, and thickest in the region of the lateral ligaments. The whitish tissue here contained irregular sinuous cavities filled with thick yellow cheesy matter. The cartilaginous surfaces of tibia and condyles of the femur were superficially destroyed, with irregular nodular surfaces in which islands of denuded carious bone were surrounded by fibrous adhesions between the tibia and condyles of the femur. The adhesions were broken by forced flexion. The patella was united with partial anchylosis to the intercondyloid fossa of femur. It was separated by chisel and hammer. The supra patellar bursa was obliterated. It was dissected loose to permit the anterior flap being held away from the condyles. A disc 2 centimeters thick was sawn from the condyles of the femur, leaving healthy cancellated structure.

The tibia was separated at its upper posterior margin from its periosteum, leaving this in connection with the posterior wall of the capsule, and a disk 1½ centimeters thick was removed. In the external half of this cut surface was an island of firm cartilaginous tissue of the epiphyseal cartilage; the cancellous surface was healthy.

On bringing the cut surfaces together the leg was straight, as in the highest degree of extension. As we desire anchylosis in a slight degree of flexion, another and wedge shaped piece was cut from the tibia, when the position was perfect. The patella was removed subperiostially by the gouge. The whitish firm tissue of the capsule was dissected and extirpated. The holes were drilled in the sides of the bones and the silver wires inserted. After removal of the elastic compression one small artery was ligated with catgut, all further bleeding was controlled by the carbolized solution. The posterior drainage tube was inserted and on a suggestion of Dr. Isham was brought out through an opening cut posterior to the hamstring tendons. The bones were brought together and the silver wires twisted; the leg placed in the splint, the anterior drainage tube inserted; the patellar ligament united; the external wound closed with carbolized silk; the drainage tubes stitched to the skin ; the leg lifted out of the splint and a perfect Lister dressing applied ; the leg replaced and the knee surrounded with oakum and fixed by a woollen bandage around the splint. The patient was put to bed and the limb suspended to a wooden frame upon the bed at its lower half.

Microscopical examination of the thickened capsule, hardened in chromic acid solution, revealed miliary tubercules throughout. (See paper in Chicago Medical Journal and Examiner, May, 1880, fig. 4.)

*Diagnosis: Miliary tuberculous arthritis of the knee-joint.* Sinuses with cheesy matter in each side of the thickened capsule, superficial tuberculous destruction—caries—of the articular surfaces of the bones. Anchylosis of the patella, progressive anchylosis and contraction of the joint. Destruction of the internal lateral ligaments and knock-knee.

The after-treatment showed the following courses :

August 6.—Some pain in knee and heel. Cotton placed between tendo Achillis and splint to relieve heel, pain ceased. Ice bag to knee. Morphia hypodermically.

August 7.—Slept two hours last night. No pain. Retention of urine. Catheter. Urine is normal.

August 8.—Catheter twice. Ammoniacal odor of urine, alkaline reaction and some pus and blood by microscope. Dressed the knee. No swelling or redness. Drainage tubes full of clots of blood. Removed by passing soft carbolized bougie through them. Fluid injected comes through, slightly bloody but without pus. (This fluid is equal parts of two and one-half per cent. solution carbolic acid and water that has been boiled.) Dressing nearly painless. To take camphor and opium pills and infusion of triticum repens.

August 9. Slept all night, Passed urine with pain at end of act. Had beef tea and toast.

August 10.—Dressed knee. Hardly any discharge from tubes. Urinary troubles continue. Washed out bladder with solution of boracic acid.

August 11.—Some pain in knee. Urine alkaline.

August 14.—Passed urine three times yesterday. Slept well. Little pain. Appetite fair. Dressed the knee.

August 18.—Dressing renewed again. No pain.

August 20.—Dressed. Upper drainage tube removed.

August 26.—Dressed. By moving limb, some union appears to have taken place between the bone surfaces.

August 28.—Pulse 96, temperature 103¾. No pain; feels well in spite of the fever.

August 29·—Dressed. *Solid osseus union.* Silver wires removed.

August 31.—A small abscess has formed by side of medial end of upper drainage tube. Incision and discharge of half a teaspoonful of thin slimy pus.

September 5, a. m.—Pulse 112, temperature 104° F. Sensation of cold followed by heat this morning. Thirst. Pain in left side, and dyspnœa. Pain is in region of ninth to twelfth ribs; pressure here painful. Physical examination gives no signs of inflammation of lung or spleen. Bowels not moved for forty-eight hours. Poultice to region of spleen. Quinine and wine and an enema.

September 6, a. m.—Pulse 120, temperature 102° F Pain less. Anorexia. Obstinate vomiting. Carbolic acid, one drop, every two hours. Champagne and ice. P. m.—Pulse 92, temperature 102.5° F. Vomiting stopped. Pain less.

September 7—Pulse 74, temperature 99.5° F. Slept well. Vomited only once. Pain nearly gone. Bowels moved twice yesterday. Feels well except uneasiness in abdomen.

September 8.—No pain or vomiting.

September 12.—*Out of bed on crutches, and with high sole on shoe of sound foot.*

September 15.—A fluctuating swelling on external side knee, round outer side hamstring tendons. Incision and escape of one ounce viscid yellow pus.

September 27.—In and about this abscess and adjoining part of the scar is found soft, bluish-red nodules of granulation tissue surrounding small sinuses that continue to discharge a little slimy purulent fluid, they show no tendency to healing. Patient anæsthetized and sinuses scraped out with sharp spoon, also the wall of the abscess—all under the spray.

October 1.—Wound healing. Patient looks a little pale. Ordered cod liver oil and syrup of iodide of iron.

October 9.—On the inner side of the knee where the drainage tubes and silver wire found exit, are some bluish-red œdematous excrescences of tuberculous granulations secreting a slimy fluid. These are cauterized with nitrate of silver.

October 13.—*Patient walking about all day. Can walk without crutches or cane.* The wound from the scraping out on outer side of the knee, all closed up.

October 17.—The granulations on inner side knee show no signs of healing in spite of repeated cauterizations. Patient anæsthetized and the tuberculous granulations removed by gouge and sharp spoon. A sinus was found leading along up and above the scar of the primary wound 3 centimeters. Wall of the sinus scraped out and drainage tube inserted.

October 25.—Opened small abscess at middle of original semilunar incision.

November 1.—The small wound healing rapidly.

November 3.—Opened another small abscess (size of hazelnut) in the interior part of scar. Patient walks about all day without pain.

In January 1880 a similar small abscess formed in the anterior part of the scar.

In February 1880 patient left hospital and began work as cutter in glove factory, being able to be about constantly.

In April a small superficial abscess again formed on the outer side of the knee. It was opened and healed in 8 days.

The present condition is as follows .

FIG. 2.

Solid anchylosis in slightly flexed position. The transverse scar irregular from abscesses, ulcers and their removal. Shortening of the extremity $1\frac{1}{2}$ to 2 inches; from the anterior superior spine of the ilium to the external malleolus, $1\frac{1}{2}$ inches and to the internal malleolus, 2 inches shorter than opposite limb. Perfectly useful limb; can stand on it all day without pain. No trace of tuberculosis apparent in other organs of the body.

A speedy osseous union is the most important point. This secured and a final success is almost certain. In our case this occurred in the third week and was complete in twenty-four days. It occurred as rapidly as in uncomplicated fractures frequently. It was a perfect instance of healing by first intention and may be considered rare in its rapidity.

But it is obvious that if we can convert an excised knee-joint into a subcutaneous fracture by antiseptic methods scrupulously carried out and immobilization, we may make a rule of what has been the exception. It might be objected that such rapid healing would not occur in advanced years as in youth, as it is known that the danger of open fractures increases with the age. But Volkmann has shown by one hundred cases of compound comminuted fractures treated antiseptically that such recover as well and rapidly as simple fractures, and regardless of the age of patient. Formerly 20 to 30 per cent. of such cases were lost by suppuration and pyæmia, now none of them provided they are treated antiseptically from the first. In excisions of the knee-joint we have the right to expect as good results under the Lister treatment as in these fractures, even better, as the wounds in the excisions are not for one moment out of the antiseptic atmosphere.

As to danger to the general health from confinement in bed, our patient was out of bed on the 38th day. Twice this length of time would do no injury.

The only accidental complication in the after-treatment was the cystitis, and that was probably due to the use of an undisinfected catheter to draw the urine during the early retention, that is so common after an operation.

There was very little fever early, and some of this may have been due to the cystitis. It was not till after the bony union that some sudden rises in temperature occurred. These were due to the formation of the small acute abscesses. The tuberculous character of the fungous arthritis in our case was the cause of the only troublesome disturbance during the after-treatment, namely, the formation of abscesses, and these were neither dangerous nor serious. The primary incision healed by first intention entirely and nowhere reopened. But about this scar the little abscesses of the size of a hazlenut already described, formed with slimy, clear fluid and slimy, viscid pus. This process is due to excessive formation of miliary tubercles in œdematous adenoid tissue, thus breaking down in fatty degeneration, making small tuberculous vomicæ, and leaving tuberculous ulcers with little or no tendency to heal. This process is unaccompanied by fever or much local pain. Sometimes about such tuberculous foci are formed small abscesses in the connective tissue.

One such formed in this case along the inner hamstrings and opened on the 31st Aug. Another, on the outer side, opened Sep. 15. Such abscesses cause slight fever as occured here. They may close up speedily and entirely, or partially and leave a small tuberculous cavity. This, as well as the bluish swollen tuberculous, ulcerated or broken down nodules, resist all local treatment. The only effective treatment is entire removal. The wound will heal rapidly and with a permanent scar.

The cause of these secondary multiple but local eruptions of miliary tubercles is obvious. Particles of the thickened fibrous capsule of the joint with their miliary tubercles have been left here and there, and now form starting points for other tubercles in soft, young adenomatous tissue. A careful removal of all the capsule is the only way to prevent these little troubles in the after treatment. This tendency of tubercles to grow in the scar should be a warning to us to remove all traces of such formation from the bone if deeper local foci are found here.

We are inclined to believe that those who hold the removal of all the capsule to be unnecessary have gathered their experience from cases of white swelling or chronic inflammation of the knee-joint with few or no deposits of miliary tubercle.

*Tuberculosis of Elbow-joint. Excision by Dr. E. W. Lee, Chicago.*

SYNOPSIS.—*Primary osteo-tuberculosis of the head of the radius. Secondary tuberculous fungous arthritis tending to anchylosis. No abscesses or sinuses. Subperiosteal excision. Recovery with joint movable and useful. Almost complete reformation of the removed osseous parts with formation of new joint.*

Mary C. B., aged 8 years. Had whooping cough at four, scarlet fever at seven, and measles a few months later. Recovery perfect. No tuberculous history. She is one of nine children; all the rest healthy and none of them ever had scrofulous disease.

At one year of age she fell down stairs—only five or six steps. Three months later her mother noticed she kept the right elbow flexed at an angle of 45 degrees; it was somewhat stiff but not swollen. The arm remained in this condition six years. No pain was experienced and no attempt was made to move the joint. Dr. Lee first saw the child when she was seven years old. Then the joint was stiff, the member was carried in a drooping manner as though it was a useless appendage. There was considerable atrophy of the muscles of the whole limb, but most of the extensors of the forearm. No active motion was possible of the elbow joint; neither flexion and extension nor pronation and supination of the forearm. By forced and very painful passive motion the forearm could be flexed 10 degrees.

The child was anæsthetized and forcible movement of the joint in all directions made, to break up adhesions. The arm was then placed in a flexible splint with rubber bands. Passive motion was now made daily and the child was induced to lift sand-bag weights. In three months she had motion of 25 to 30 degrees. Soon thereafter the joint became swollen and tender and passive motion had to be discontinued. The joint soon became as stiff as at first.

Passive motion was resumed on two occasions later but without benefit. Each attempt was followed by pain and swelling.

Subperiosteal excision was made January 27, 1880, at the patient's home, by Dr. Lee, assisted by Drs. Fenger and Bridge. The patient was etherized and Esmarch's bandage applied. Then, under the spray, a longitudinal incision eight centimeters long was made on the posterior side of the joint, dividing the tendon of the triceps longitudinally. The periosteum was detached by a strong gouge, thus preserving the attachment of the extensor tendons to the periosteal sac of the olecranon.

In the same way the head of the radius and the cubital epiphysis of the humerus were enucleated, leaving their periosteal covering in connection with the overlying soft tissues. In the cavity of the joint were a few drops of slimy, grayish pus. The articular cartilages were destroyed and the bones presented partly rough denuded surfaces, partly a soft, reddish-gray covering consisting of fungous granulations.

The olecranon was removed entire, also the head of the radius. Of the lower end of the humerus two centimeters in length was sawed off. The cut surfaces of bone were healthy; one small artery only was ligatured and that with catgut. A drainage tube was inserted the whole length of the wound, which was closed with silk sutures. The Lister dressing was applied and the arm fixed to an angular splint. Examination of the excised bone showed that the head of the radius had lost its cartilaginous covering on the articulating surfaces and presented several small cavities or excavations two to three millimeters deep and the same in diameter. The walls and base of these cavities consisted of white, hard and smooth osseous tissue with no fungous granulations on the surface. The shape of the cavities indicated that probably here a primary osteo-tuberculosis had developed, at first in the spongy portion of the bone. These tuberculous foci had enlarged and finally reached the articular surface, opened into the cavity of the joint and caused the fungous tuberculous arthritis. The osteo-tuberculosis in the head of the radius has come to an end, the cheesy matter and the lining membrane of adenoid tissue and miliary tubercles have disappeared and left a clean cavity with no further tendency to destruction of its walls.

The articular epiphysis of the humerus presented a different stage or form of the disease. Here we find the secondary, superficial, diffuse osteo-tuberculosis or tuberculous caries destroying the bone from the surface. The cartilages were all gone and the articular extremity of the bone was altered in shape and reduced in size. In some places rough, bony surfaces were present, in others irregular layers of reddish-gray fungous granulations covering the bone. The tissue was so friable as to be easily removed from the bone, which presented then a roughened surface. The miscroscope showed these granulations to be adenoid tissue containing spiculæ of bone undergoing absorption and miliary tubercles. The articular surface of the olecranon showed the same morbid conditions as the humerus.

The after-treatment was fortunate. No suppuration occured. The wound healed by first intention. Drainage tubes were removed in two weeks. Dressings were renewed every two or three days. In three weeks the wound was entirely healed.

After four days, passive motion was commenced with the screw of the splint. After 10 days, passive motion was made at each dressing, the splint being removed.

After four weeks the splint was dispensed with and active motion commenced. Later, to force the child to use the arm, the well arm was tied to the waist and kept beneath the clothing.

She now has a very useful arm with new formed joint—as the members may see by examining the patient. There is 90 degrees of motion without pain, i. e. flexion and extension. She uses the arm for everything, and the whole day long without pain or fatigue.

There is shortening of the whole extremity by about three centimeters—two of which are due to shortening of the humerus.

In examining the new formed joint we easily feel a large new formed olecranon and condyles of the humerus, the external the largest.

Pronation and supination are permitted to about half the normal extent, viz., to 80 and 90 degrees. There is a slightly lessened volume of muscle of the whole extremity, amounting for the arm to three-quarters of an inch in circumference, and for the forearm to one-half inch.

The favorable course and result of the case, absence of fever and suppuration may be due to the antiseptic methods used. The splendid result as to mobility of joint is due of course to the subperiosteal method of operating.

We need hardly say that the older methods with destruction of periosteum are never to be employed where mobility of joint is expected afterward.

Will the good result as to motion of this joint be lasting?

For elbow and shoulder joints we know the results may be good several months after the operation, and later all active motion be lost either from anchylosis due to relapse of the fungous arthritis, or by relaxation of the tendons and ligaments of the new formed joint, the latter producing what may be termed a loose joint.

We hope to show this patient to the society again a year hence when the permanent result of the case may have been reached.

*Tuberculosis of Hip-joint. Excision by Dr. E. W. Lee, of Chicago.*

Before relating the history of this case we wish to say that we are unable to exhibit the patient as she is still under treatment.

The case is communicated to illustrate some improvements in bandaging and dressing the patient after this operation, and because the tuberculous character of the disease in this case has brought out a measure of importance in the after-treatment.

SYNOPSIS.—*Tuberculous fungous arthritis, caries, morbus coxarius of right hip-joint of three years standing. Large anterior abscess. Excision subperiosteal by external longitudenal incision; the abscess emptied and scraped out from the incision. Drainage through two openings. Three months later the inclosed abscess again laid open by a large incision and its entire wall of tuberculous tissue scraped out, the abscess closing entirely in eight days thereafter. The leg dressed in Sawyer's modification of Hodgen's splint. The dressings re-applied as often as required by means of Lee's frame for the dressing of excisions of the hip-joint.*

Sarah Jane B., aged 9½ years. No consumption, cancer or hip disease in family. The father's parents both living. Mother's father died in her infancy of some disease unknown; her mother living. The patient is one of a family of ten children, of which five are living and healthy; one died of cramps, one of teething, one of diphtheria and one was still-born. No scrofula or skin disease has occured to any of the family. She had whooping cough and varicella at five years of age and measles at seven.

The hip disease began Feb. 1877. Eight months previously she had slipped down a short flight of stairs (3 or 4) but complained of no pain afterward, and was not lame for six months. Then symptoms began. She was taken to Dr. Gunn who recognized the hip disease and put her under treatment in St. Luke's Hospital and afterward at home. The treatment was by splints and extension by weight and pully and was continued two months.

She improved, could walk and run and jump without appearance of lameness.

In the winter of 1878-9 she fell from a chair upon her back upon the floor. She appeared only slightly hurt but rapidly grew lame. Six months later she was so bad she was obliged to use crutches. Early in December 1879, the mother noticed a hard swelling anterior to the diseased joint. She consulted Dr. Lee who pronounced it an abscess originating from a carious hip-joint and advised excision.

Operation by Dr. Lee was performed December 24, '79 with the assistance of Drs. Fenger, Isham, Clarke, Bridge and McLennan. At the time of operation the child was poorly nourished, and had hectic. The thoracic and abdominal organs were normal. The right lower limb was shortened 1½ inches, adducted and flexed. Active movements of the hip-joint were impossible on account of pain. Under the influence of ether, limited motion was possible, but no distinct crepitus was produced. A large fluctuating abscess was felt reaching from the anterior superior spine of the ilium down to the upper third of the femur anteriorly.

Under the carbolic spray a longitudinal incision was made from a point five centimeters above, down along the outside of the trochanter major five cms. The periosteum was detached from the bone, the capsule opened and the head dislocated from the acitabulum. As the head of the bone was atropied from superficial caries and the neck presented a tuberculous cavity (as described in a previous paper where it is shown that the cavity reached down to the lesser trochanter), the upper extremity of the femur was removed just below the lesser trochanter. The cut surface of the femur was here healthy. The acetabulum presented several roughened surfaces, partly covered with tuberculous granulations. These were gouged out until firm osseous substance was met with. The anterior abscess was opened through the wound and its wall scraped off and the membrane removed in pieces. A counter opening was then made at the lower extremity of the abscess and a drainage tube inserted through its whole length and fastened with sutures.

The acetabulum was washed out with a 2½ per cent. solution of carbolic acid, and two drainage tubes inserted and fixed to the respective ends of the incision with sutures when the wound was closed with silk sutures.

A perfect Lister dressing was applied, over which was placed a layer of carbolized oakum, and the patient was placed into Sawyer's splint.

This new and splendid apparatus for dressing excised hip-joints, combining extension and suspension of the operated extremity, was first applied in the summer of 1879 in Cook County Hospital, Chicago, on a patient operated upon by Dr. Fenger.

We here present drawings of the instrument and the written description of the apparatus, prepared by Dr. Sawyer himself.

The splint was a modification, or rather an extension of the well known Hodgens' anterior splint, employed in fractures of the femur, etc. The outlining framework was constructed of five-eighths inch iron, while the cross-bars arching over the limb and body, as hereafter described, were of three-eighths inch iron.

The five-eighths iron bar constituting the main framework of the splint, commenced at the lower border of the axillary space on the side of the affected limb and was extended downwards to a point six inches below the foot, being moulded so as to correspond, with approximate accuracy, to the outline of the body. Below the foot the bar made a square turn, extending horizontally inward about four inches; thence turning squarely again and running up the inner aspect of the affected limb to the groin. From this point it arched across the opposite groin in an oblique direction corresponding with the inguinal fold and at the anterior superior spinous process of the opposite ilium, took another and final turn, ascending along the opposite side of the body to its termination at the lower border of the axillary space. Arching across the limb and body, and connecting the two branches or "legs" of this framework, were five cross pieces at the following points: first, at the upper extremity of the splint; second, extending obliquely from a point six inches below the upper extremity on the affected side to the angle of the splint corresponding to the anterior superior spinous process of the opposite side; third, at a point corresponding to the inner and lower extremity of the fixed arch across the opposite groin; fourth, at the lower third of the thigh; and fifth, at the lower third of the leg. Of these arches all but the third terminated on either side in a hook for the purpose of suspending the apparatus. There was a slight bend in the splint corresponding to the knee-joint, so as to allow of limited flexion, and another at the upper end of the femur, where it was bent upward at an angle of perhaps 20°.

This apparatus was applied after the manner of a Hodgens splint. Adhesive straps were applied to the limb as high up as the lower third of the thigh, being secured to the limb by a roller. These straps were attached at their lower extremities to a "stirrup block," which was in turn fixed to the lower end of the splint by means of a short piece of elastic tubing.

The limb and body were supported by broad strips of muslin passing underneath and secured by pins on either side to the framework of the splint.

The apparatus was suspended by two sets of cords as follows: 1. Four being converged from the four lower hooks already mentioned to a point some distance above the limb, were attached to a line dropped from a pully in the ceiling a little below the foot of the bed, the degree of obliquity in the direction of this line varying with the amount of extension desired. 2. Four cords converging from the four upper hooks to a point a few inches above the body, were attached to a line dropped directly downward from the ceiling, a set of compound pulleys intervening for convenience in elevating the body. For convenience in dressing the limb, a block was removed from the mattress corresponding to the location of the wound. This could be replaced when not dressing.

FIG. 3 shows the iron frame of Sawyer's splint.

Dr. Sawyer's splint proved in this first case—a boy of seventeen years of age—a most excellent and convenient help in the after-treatment. When the dressing was renewed the patient hoisted himself with the upper system of pulleys, an assistant raising the limb by the lower set of pulleys, the straps covering the wound were removed after drawing the block in the mattress and the wound easily gotten at.

An important point in the after-treatment, if we would carry out all the details of the antiseptic method, is this: We must be in no hurry, but must have ample time to cleanse every part of the wound, wash out the tubes, etc., without fear of tiring the

FIG. 4 shows the splint applied on the excised hip.

patient or causing him to get nervous or suffer pain. Therefore it is necessary to have the whole body of the patient easily suspended and high enough to enable the surgeon to get free access

FIG. 5.

to the wound from below, to operate the atomizer and to change the bed clothes. To accomplish this, Dr. Lee devised a movable suspension frame with straps, to support the patient during the shifting of the dressing.

Fig. 5 shows Lee's frame for dressing of excisions of hip-joint. It consists of a wooden frame six feet long, two and a half feet wide, with cross straps four inches wide and four inches apart. Each corner has a strong iron hook for attaching a suspension rope.

The center of each of the end pieces bears another hook for fixing the end of the rope after the apparatus is hoisted.

The mode of operating is as follows: The patient is first hoisted in Sawyer's splint five to ten inches above the bed. Lee's frame is placed on the bed beneath, when the patient is lowered upon it. The ropes are then adjusted to the frame and this is raised up two feet, bearing patient, Sawyer's splint and all, and fixed in this position. The patient has a pillow under his head and is perfectly comfortable.

The bed is then taken away. The straps in Sawyer's splint covering the Lister dressing are unbuttoned and removed. Next the straps in Lee's frame covering the wound are removed. The antiseptic dressing is then accessible, is easily removed and the wound cleansed, under the spray. The bed is refreshed and the new antiseptic dressing in all its layers is prepared and laid upon the bed in the proper place, when the bed is brought under the patient and he is let down upon the dressing, the application of which is completed by bringing around the body the ends of the many-tailed bandage that holds the dressing firmly to the part.

The straps of the splint are readjusted and the patient hoisted by this away from the wood frame which is now removed, and the patient is again lowered on the bed and the dressing is finished.

The description of this dressing and the apparatus may make it appear complicated and unpractical. But if you could see the dressing made upon these little patients and see them, as we often have seen them, laugh, chatter and make fun during the whole of the tedious performance, which otherwise must be tiresome and painful, you would not entertain such an opinion of it.

The splints and dressing hitherto in use for the after-treatment of excisions of the hip-joint can none of them be used when a perfect Lister dressing is to be made, without the greatest inconvenience. The plaster of Paris bandages of the Germans, although they may be combined with both extension and suspension are not strong enough when sufficiently large fenestræ are cut for changing the dressings and they cannot be kept clean and their renewal is attended with great inconvenience and discomfort to the patient. The best apparatus hitherto used is, in our opinion, Sayre's wire apparatus or splints. (Sayre's Orthopedic Surgery and Diseases of Joints, N. Y., 1879.) This combines immobilization and extension. It does not permit as free access to the wonnd for the application of the Lister dressing as our own apparatus, neither can it be kept as clean; furthermore it is much more expensive.

Volkmann, in a valuable monograph in 1873 (Die Resectionen der Gelenke, Sammlung Klinischer Vorträge, N. 51, 1873, p. 306), made the following remarks: "I do not deny that the invention of a, so to speak, mobile, immovable apparatus for dressing excised hip-joints, whereby cleansing the wound, defecation, etc., can be accomplished without moving the patient, would be a great advantage."

The apparatus already described,

1. Permits defecation, dressing the wound, changing the bed clothes, etc., without moving the patient; it lifts the patient without moving him.
2. It can easily be kept perfectly clean.
3. It is inexpensive.

We regard it as a step toward the accomplishment of the desideratum of Volkmann.

COURSE OF THE AFTER-TREATMENT.

| | | | | | |
|---|---|---|---|---|---|
| December | 25, | Pulse | 140 | temperature | $102\frac{1}{5}$ Fahrenheit. |
| " | 26, | " | 136 | " | $102\frac{2}{5}$ |
| " | 27, | " | 138 | " | 103 |
| " | 28, | " | 130 | " | $102\frac{3}{5}$ |
| " | 29, | | | " | 101 |
| " | 30, | | | " | 101 |

December 31, Temperature 101.

January 1, Temperature 101.

January 3, Temperature 103.

January 4.—On making the dressing the inferior opening into the abscess is completely plugged by a mass of tissue. On pulling it out it was found to be as large as a hen's egg and consisted of tuberculo-adenoid tissue of the membrane lining the abscess.

January 5.—The temperature had fallen to 102.

Thereafter the temperature steadily fell, and the case progressed favorably.

In 2½ months the hip had become so firm that the child could be moved in any way without pain. The Sawyer's splint was then dispensed with and ordinary extension by weight and pulleys substituted.

Three weeks later the child had an attack of facial erysipelas —the patient lived in a deep basement, and something of an epidemic of erisipelas was then going on. Only the head, neck and shoulders were involved, and the attack lasted two weeks.

The erysypelas had no effect on the wound. During its course however the precaution was taken to dress the case oftener,—viz., every two instead of every four days.

After convalescence from the erysipelas was established, the granulation tissue in the lower opening of the abscess broke down and the discharge increased in quantity and continued in spite of antiseptic precautions. As the granulation tissue of the now almost closed excision wound showed symptoms of breaking down, it was deemed advisable to extirpate the walls of the abscess.

This operation was made March 30 under anæsthesia. The abscess was freely laid open and was found to communicate by a sinus with the excision wound. This sinus extended to a denuded osseous surface above the upper posterior border of the acetabulum on the iliac bone. The upper end of the femur and the acetabulum were united and surrounded by a firm connective tissue mass in which no sinuses or traces of inflammation could be found.

The abscess was lined with a grayish soft, friable but adherent membrane, one to three millimeters in thickness, with smooth surface and without visible miliary tubercles. This mass was entirely scraped off down to healthy fibrous and muscular tissue. The denuded osseous surface was also scraped out. The hæmorrhage was insignificant. Two drainage tubes were inserted, one along the whole length of the abscess, the other reaching to the denuded bone and being brought out through the old excision opening. Microscopical examination showed the excised membrane to consist of adenoid tissue and thousands of miliary tubercles.

On April 1st the Lister dressing was reapplied and no pus flowed from the abscess.

April 3.—Some pus came out the upper posterior drainage tube. In one week the tube was removed from the abscess, and in three days the sinus left by the tube had closed.

Two days later the second tube was removed and now a little sinus is left leading to the denuded bone.

The second point to which we wish to call special attention in the treatment of this case is the careful removal of the tuberculous lining of the abscess.

As Volkmann has advised, these abscesses should be laid open throughout their whole length. If in our case this had been done at the first operation probably its repetition would not have been called for. We feared at first to make this thorough operation on account of the large external wound it required, and so our first scraping out was not effectual.

As experience has shown that the external wound independently of its size, as well as the wall of the abscess, closes by first intention when the tuberculous matter is all removed and the operation is done antisiptically, we need not fear to make free and large incisions.

Such an operation as this is the only means we have of closing tuberculous abscesses and of saving our patients from the dangers incident to exhausting suppuration and in time amyloid degeneration of kidneys, spleen and liver, and from general tuberculous infection.

## Figures and Tables

**Fig. 1. f1:**
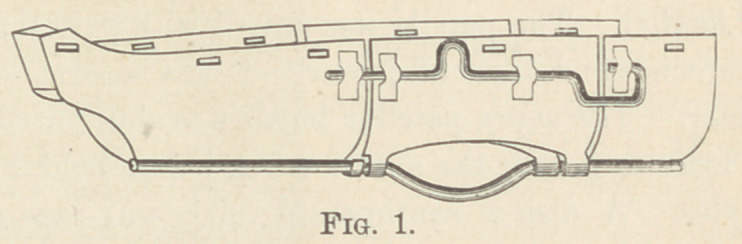


**Figure f2:**
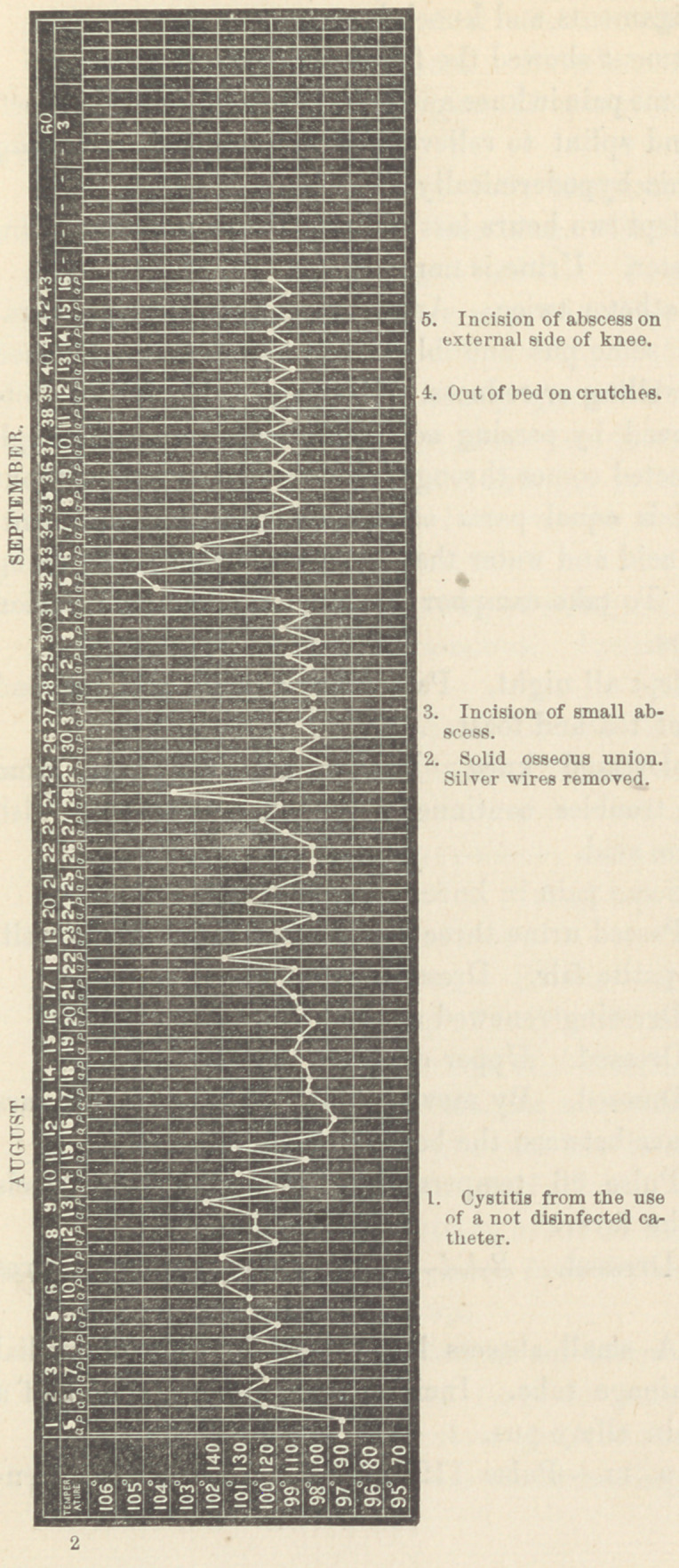


**Fig. 2. f3:**
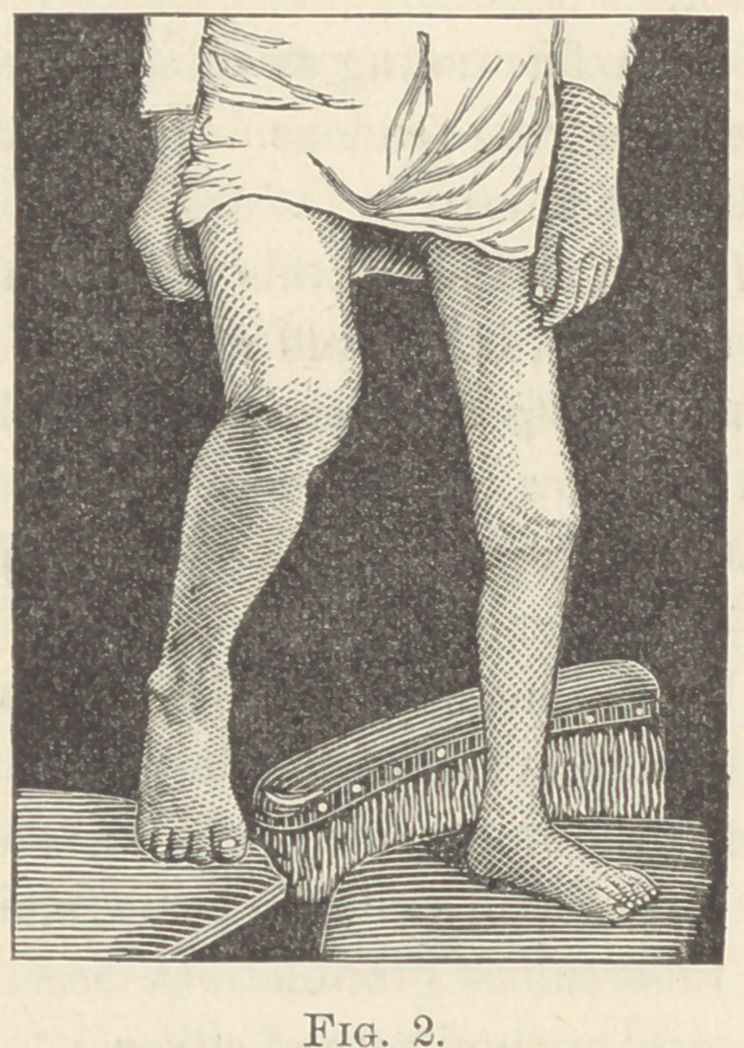


**Fig. 3 f4:**
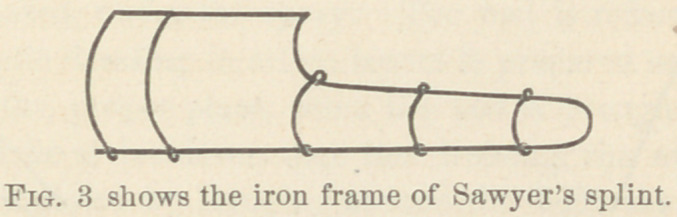


**Fig. 4 f5:**
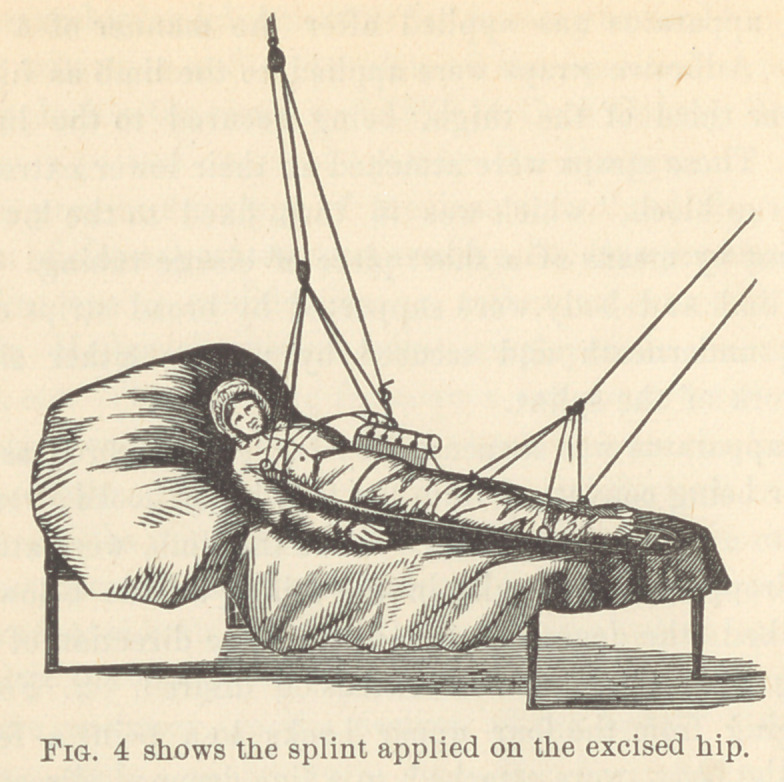


**Fig. 5. f6:**